# Kallikrein family proteases KLK6 and KLK7 are potential early detection and diagnostic biomarkers for serous and papillary serous ovarian cancer subtypes

**DOI:** 10.1186/s13048-014-0109-z

**Published:** 2014-12-05

**Authors:** Ayala Tamir, Ushma Jag, Sreeja Sarojini, Craig Schindewolf, Takemi Tanaka, Rajendra Gharbaran, Hiren Patel, Anil Sood, Wei Hu, Ruzeen Patwa, Patrick Blake, Polina Chirina, Jin Oh Jeong, Heejin Lim, Andre Goy, Andrew Pecora, K Stephen Suh

**Affiliations:** The Genomics and Biomarkers Program, The John Theurer Cancer Center, Hackensack University Medical Center, D. Jurist Research Building, 40 Prospect Avenue, Hackensack, NJ 07601 USA; Thomas Jefferson University, Philadelphia, PA 19107 USA; Departments of Gynecologic Oncology and Cancer Biology, MD Anderson Cancer Center, University of Texas, Houston, TX 77030 USA; Sophic Systems Alliance, Inc, Rockville, MD 20850 USA

**Keywords:** Biomarker, Ovarian cancer, Diagnostic, Early detection, Bioinformatics

## Abstract

**Background:**

Early detection of ovarian cancer remains a challenge due to widespread metastases and a lack of biomarkers for early-stage disease. This study was conducted to identify relevant biomarkers for both laparoscopic and serum diagnostics in ovarian cancer.

**Methods:**

Bioinformatics analysis and expression screening in ovarian cancer cell lines were employed. Selected biomarkers were further validated in bio-specimens of diverse cancer types and ovarian cancer subtypes. For non-invasive detection, biomarker proteins were evaluated in serum samples from ovarian cancer patients.

**Results:**

Two kallikrein (KLK) serine protease family members (KLK6 and KLK7) were found to be significantly overexpressed relative to normal controls in most of the ovarian cancer cell lines examined. Overexpression of *KLK6* and *KLK7* mRNA was specific to ovarian cancer, in particular to serous and papillary serous subtypes. *In situ* hybridization and histopathology further confirmed significantly elevated levels of *KLK6* and *KLK7* mRNA and proteins in tissue epithelium and a lack of expression in neighboring stroma. Lastly, KLK6 and KLK7 protein levels were significantly elevated in serum samples from serous and papillary serous subtypes in the early stages of ovarian cancer, and therefore could potentially decrease the high “false negative” rates found in the same patients with the common ovarian cancer biomarkers human epididymis protein 4 (HE4) and cancer antigen 125 (CA-125).

**Conclusion:**

KLK6 and KLK7 mRNA and protein overexpression is directly associated with early-stage ovarian tumors and can be measured in patient tissue and serum samples. Assays based on KLK6 and KLK7 expression may provide specific and sensitive information for early detection of ovarian cancer.

**Electronic supplementary material:**

The online version of this article (doi:10.1186/s13048-014-0109-z) contains supplementary material, which is available to authorized users.

## Introduction

Ovarian cancer (OVC) ranks as the fifth most common cancer in women and has the highest mortality rate among gynecologic malignancies [1,2]. Although the 5-year survival rate of OVC is around 90% when detected in its early stages (I/II), nearly 80% of new cases are diagnosed in advanced stages (III/IV) because of the asymptomatic nature of the disease. Unfortunately, the 5-year survival rate of advanced OVC is as low as 11% [3].

Identification of early detection biomarkers for ovarian carcinoma remains a challenge due to a wide range of morphological, clinical and genetic variations found in OVC progression [4]. Currently available biomarkers lack specificity and sensitivity that are required for routine clinical use [5]. The only clinically validated biomarker used for early detection, disease monitoring, and assessing relapse or response to treatment is cancer antigen 125 (CA-125 or MUC-16). Although CA-125 serum expression is elevated above normal levels in early stage (23%) and late stage (80%) disease, this antigen lacks specificity and sensitivity for use as a single marker for early OVC detection [6,7]. In addition, CA-125 overexpression is frequently observed in benign conditions (e.g., endometriosis) and thus it lacks accurate diagnostic value for early stage disease [8]. As an early detection biomarker of OVC, recent reports suggested that the WAP four-disulfide core domain 2 (WFDC2 or HE4) protein could provide greater sensitivity and specificity than CA-125 [9,10]. An assay that detects a combination of HE4, CA-125, carcino-embryonic antigen-related cell adhesion molecule 5 (CEACAM5) and vascular cell adhesion molecule 1 (VCAM1) expression in serum has demonstrated significantly better sensitivity for early stage OVC detection compared to benign tumors [11,12]. In addition, the Food and Drug Administration (FDA) has approved the OVA1 test, which consists of a panel of five biomarkers: transthyretin, apolipoprotein A-1, beta2-microglobulin, transferrin and CA-125 [13]. Slightly after the approval of the OVA1 test, the FDA approved the use of the Risk of Ovarian Malignancy Algorithm (ROMA) that involves CA-125 and HE4 measurements and a definition of menopausal status [14]. These tests suggest that use of a combination of multiple markers can generate synergistic advantages over single marker diagnostics [15].

A number of human kallikrein-related peptidase (hKLK) family members have been associated with human cancers and exhibit differential expression in many types of advanced cancers, including gastrointestinal, head and neck, lung, ovarian and brain [16-18]. However, to date few reports have focused on detection of early stage OVC using KLK markers, or more specifically, by measuring levels of kallikrein-related peptidase 6 (KLK6) and kallikrein-related peptidase 7 (KLK7). In a study by El Sherbini *et al*., 40% of stage I/II ovarian cancer patients (n = 10-15) presented above normal levels of KLK6 while 83.3% of stage III/IV patients (n = 12) were KLK6 positive [19]. The hKLK family is comprised of 15 homologous secreted trypsin- or chymotrypsin-like serine proteases, encoded by tightly clustered genes found in the chromosome 19q13.4 region [20]. hKLK transcription is regulated by many stimulatory and inhibitory factors, including steroid hormones [21]. hKLKs are co-expressed in the epithelia of several organs and mediate a range of physiological functions, including skin desquamation and body fluid homeostasis [22]. A number of studies have found that hKLK genes/proteins are aberrantly expressed in multiple human cancers, and their overexpression in late stage tumors is often associated with unfavorable patient prognosis [23-25]. hKLKs are associated with cancer cell growth, angiogenesis, invasion and metastasis through their proteolysis of signaling proteins and extracellular matrix components [26], and in a recent study hKLKs were associated with elevated TGFβ signaling in ovarian cancer cells [27]. While elevated hKLK expression has been reported in late stage OVC [28,29], its expression or role in early disease stages has not been extensively studied.

In this study, we aimed to identify early detection biomarkers for OVC by using a multistep screening method of bioinformatics-guided selection and subsequent expression screening in relevant cell lines, followed by expression validation in patient samples. We were able to identify at least two biomarkers, KLK6 and KLK7, by this strategy and characterized their potential for detecting early stage OVCs. We then compared the levels of these proteins in early stage OVC patients to levels of proteins commonly used for OVC detection, including CA-125 and HE4.

## Materials and methods

### Bioinformatics

The Biomax BioXM™ Knowledge Management System (Biomax Informatics AG, Munich, Germany) was used to mine and generate a rank list of candidate OVC genes from the 6,955 manually curated cancer genes of the National Cancer Institute (NCI) and the Cancer Gene Index (CGI). The Biomax BioLT ™ Tool (NLP) was used to mine 18 million Medline abstracts (94 million sentences) to find and validate genes associated with cancer terms, gene-disease relationships and gene-compound/treatment relationships for each of the 6,955 cancer genes. The NCI Thesaurus Role Codes and Karp’s Evidence Codes [30] were used to annotate over 1.3 million related sentences. The search for potential biomarkers was performed by initiating queries on BioXM with a combination of search terms, including ovarian, cancer, biomarker, over-expression and up-regulation or down-regulation.

### Cell culture

OVC cell lines TOV21G, TOV112D, OV-90, CAOV3, SKOV3, PA-1, SW626, and ES-2 [31-38] were purchased from the American Type Culture Collection (ATCC, Manassas, VA) and cultured in the media suggested by the distributor. SKOV-1, IGROV-1, HEY, OV-2008, A2780, UCI-101 and UCI-107 cells [39-45] were obtained from Drs. Howell (University of California, San Diego) and Carpenter (University of California, Irvine), and were all cultured in RPMI 1640 medium (Invitrogen, Carlsbad, CA). The DOV13 cell line [46] was obtained from Dr. Bast (MD Anderson Cancer Center) and cultured in DMEM. The CSOC882 cells [47] were obtained from Dr. Karlan (University of California, Los Angeles) and cultured in McCoy’s 5A medium. Cell line 2774 [48] was obtained from Dr. Wolf (MD Anderson Cancer Center) and cultured in EMEM. The BG-1 cell line [49] was obtained from Dr. Korach (NIEHS, National Institutes of Health) and cultured in a 1:1 mixture of DMEM and F12 media without phenol red. The normal ovarian epithelial cell lines FHIOSE118 and IOSE523 [50,51] were obtained from Dr. Cheng (Moffitt Cancer Center) and Dr. Nelly Auersperg (University of British Columbia), respectively, and cultured in a 1:1 mixture of MCDB105 and Medium 199. All culture media were supplemented with 5-15% v/v fetal bovine serum (FBS; Hyclone, Logan, UT) and penicillin/streptomycin solution (Invitrogen). A detailed list of all ovarian cells lines used in this study is provided in Additional file 1.

### RT-PCR and statistical analysis

Total RNA was extracted from cells using Trizol (Invitrogen), and cDNA was generated with the SuperScript III RTS First-Strand cDNA Synthesis Kit (Invitrogen) as described by the manufacturer. All amplification primers were synthesized for use with the ABI7900 RT-PCR device (Applied Biosystems, Foster City, CA) as recommended by Applied Biosystems, and they were demonstrated to produce a single PCR band of the expected size by agarose gel electrophoresis of end-point PCR products from cDNA templates generated from normal ovarian cell lines. All qPCR assays used MicroAmp Fast Optical 96-Well Reaction Plates with Barcode (Applied Biosystems) in the standard mode (first denaturation at 95°C for 10 minutes, and then 40 cycles at 95°C for 15 seconds followed by 60°C for 1 minute). The qPCR data were normalized against internal GAPDH or β-actin cDNA and then analyzed by software provided with the ABI7900. Poor quality specimens that produced no meaningful values after 40 cycles of qPCR were not included in the data processing steps. The TissueScan Cancer Survey Panel and OVC Panel I-IV (both from OriGene, Rockville, MD) were used as described by the manufacturer. All cancer tissues in these panels contain an average of 75% cancer cells and 25% surrounding stroma. Ovarian tissue samples were also obtained from tissue banks at the MD Anderson Cancer Center (MDACC) and Thomas Jefferson University (TJU), which were both IRB-approved. All qRT-PCR assays were done in duplicate, and experiments were repeated a minimum of two times. For statistical analysis, SigmaPlot 12.5 (SysStat Software, Chicago, IL) or JMP4 (SAS Institute) software was used to determine p values of differences in expression between ovarian and other cancer types versus corresponding normal tissues. A t-test or analysis of variance (ANOVA) was used to calculate differences between means of sample groups versus normal controls and derive the corresponding p values.

### Immunohistochemistry

Whole-mount paraffin-embedded tissues and tissue arrays (US Biomax, Rockville, MD; Proteogenex, Culver City, CA; and the Tissue Bank of Thomas Jefferson University, IRB-approved) were subjected to histochemical staining as described by the antibody manufacturers. Diaminobenzidine (DAB) staining was visualized by brightfield microscopy. The slides were photographed with an Axio Imager Microscope (Carl Zeiss, Thornwood, NY), and images were taken at 20× and 40× magnification.

### *In situ* hybridization

Ovarian tissue sections were de-paraffinized, processed for high pH antigen retrieval, de-proteinated by proteinase K treatment (10 μg/ml) (Roche) in a 37°C water bath for 20 minutes and then treated with 0.2% w/v glycine for 30 seconds to inactivate the enzyme. After fixing the sections with 4% w/v paraformaldehyde (PFA; Electron Microscopy Sciences, Fort Washington, PA) for 10 minutes, the sections were blocked with hybridization buffer (50% v/v formamide, 5× saline-sodium citrate (SSC), 9.2 mM citric acid, 50 μg/ml heparin, 500 μg/ml yeast RNA, and 0.1% v/v Tween-20) for 2 hours at 54°C. The sections were incubated overnight in a humidified chamber with digoxigenin (DIG)-labeled probes (20 nM, Exiqon, Woburn, MA). The sequence of the *KLK6* [GenBANK: NM_001012964] probe was 5′-DIG-GACCAAGTCCTCACTCATCAC-3′, and for *KLK7* [GenBANK: NM_139277] the probe was 5′-DIG-AAAGTACACAGAAGGAAGGAGA-3′. The sections were washed for 30 minutes three times with hybridization washing solution (50% v/v formamide containing 2× SSC) at 54°C and then five times for five minutes each with washing solution (0.1% v/v Tween-20 in phosphate-buffered saline, PBS) at room temperature. After blocking for 3 hours at room temperature with 5% w/v bovine serum albumin (BSA), the sections were treated with mouse anti-DIG antibody (SC-57583, Santa Cruz Biotechnology, Santa Cruz, CA) at a dilution of 1:1000 in 5% BSA overnight. The sections were washed four times for five minutes each in PBS containing 0.1% v/v Tween-20, and then once in PBS for 5 minutes. To visualize bound probe, the Envision G/2 System/AP Rabbit/Mouse Permanent Red kit (Dako, Carpinteria, CA) was used as described by the manufacturer. The stained tissues were further processed and photographed as described above for immunohistochemistry.

### Immunoblot analysis

Serum samples from OVC patients (n = 44) and normal female donors (n = 10) were purchased from Proteogenex (Culver City, CA) and Bioserve (Beltsville, MD). The samples represented diverse tumor stages, age groups and races. 100 μl of serum was used as starting material from all samples and abundant serum proteins were depleted with ProteoPrep Blue Albumin and IgG Depletion Kit (both from Sigma-Aldrich, St. Louis, MO) as described by the manufacturer prior to SDS-PAGE. Twenty micrograms of proteins from eluates in loading buffer (0.5 M Tris–HCl, 0.15 M NaCl, 1% IGEPAL, complete mini [Roche Applied Science, Indianapolis, IN] containing 10 μg/ml leupeptin, 10 μg/ml aprotinin, 1 mM p-methylsulfonylfluoride [PMSF], 1 mM NaVO_3_, 0.05 M NaF, and 1 mM EGTA) were resolved by SDS-PAGE in 12.5% w/v gels and then further examined by western blot analysis. The membranes were probed with primary anti-KLK6 (H60) and -KLK7 (1407) antibodies (Santa Cruz Biotechnology) and then with appropriate horse radish peroxidase (HRP)-conjugated secondary antibodies (Jackson ImmunoResearch Laboratories, West Grove, PA). The reactive proteins were visualized by chemiluminescence with SuperSignal West Dura substrate (Thermo Fisher Scientific, Rockford, IL).

### Enzyme-Linked Immuno-Sorbent Assay (ELISA)

ELISA kits (R&D Systems, Minneapolis, MN) for measuring the presence of CA-125 and HE4 in corresponding OVC patient serum (Proteogenix, Culver City, CA) were used for this study and the manufacturer’s protocol was followed. Recombinant human CA-125 (R&D Systems, Minneapolis, MN) or HE4 (Novoprotein, Summit, NJ) were used as positive controls and further diluted as standards. Nineteen serum samples of early stage patients were evaluated for this purpose: Stage I (7 patients) and stage II (12 patients), and the same proteins were compared in two stage III and two stage IV patients. Levels of these proteins were also measured in serum from three healthy individuals. Serum was diluted (1:4) before the measurements and the results were calculated as averages of triplicates of each serum sample. The colorimetric results were read at 495 nm on a BioTek Synergy HT reader and Gene5 software was used to read and analyze the results.

A detailed description of the samples used (including numbers, sources and suppliers) as related to the assays performed is provided in Additional file 2.

## Results and discussion

### Pre-screening of potential OVC biomarkers using bioinformatics tools and an OVC cell line library

**Figure 1:**
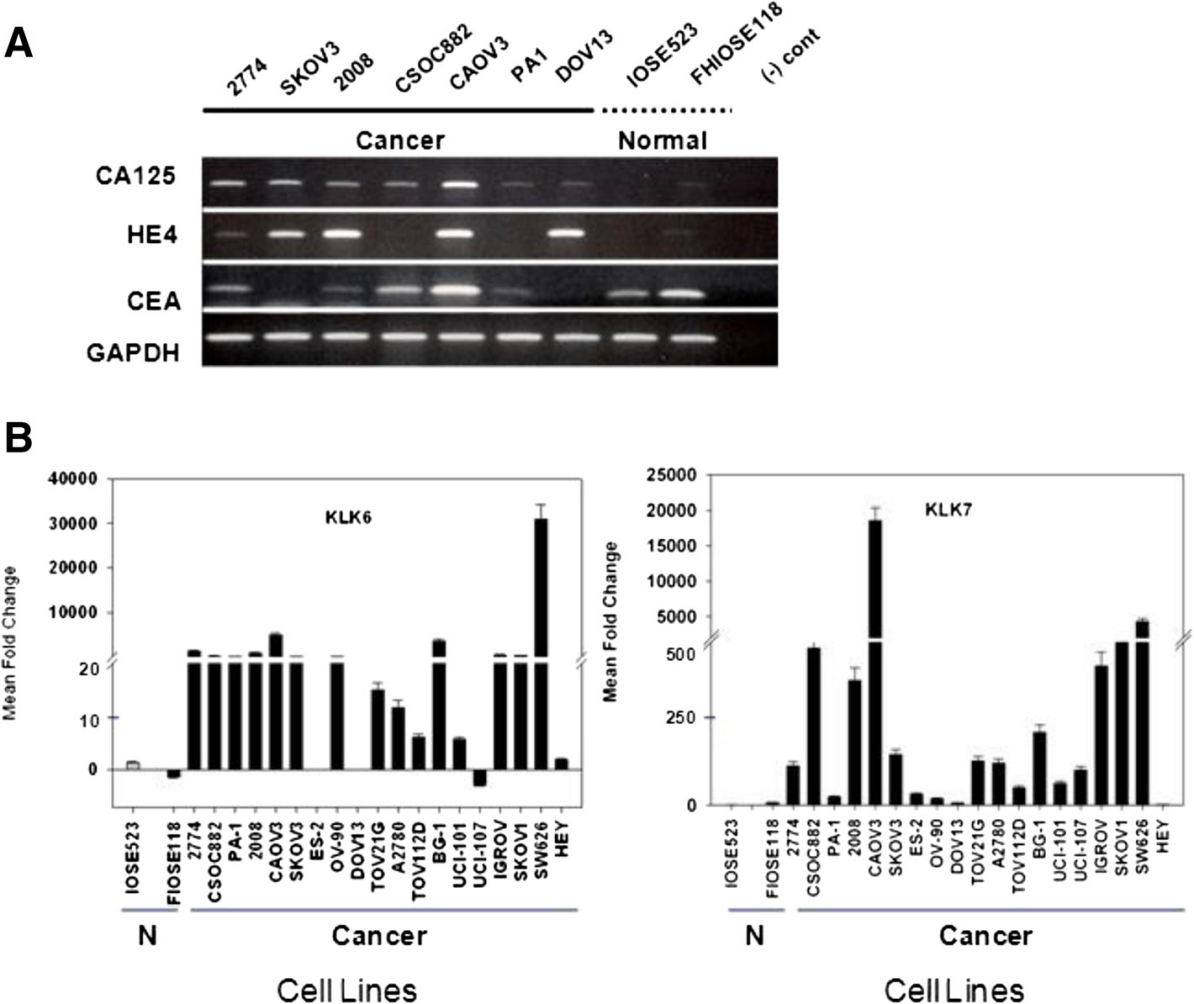
**Overexpression of KLK6 and KLK7 genes was observes in OVC cell lines. (A)** Established OVC cell lines representing different ages, stages and subtypes were selected and tested by end-point PCR for expression of known OVC genes (CA-125, HE4, and CEA) relative to normal ovarian epithelial cell lines. Amplified cDNAs were qualitatively compared following electrophoretic separation as ethidium bromide-stained bands on agarose gels. **(B)** Gene expression of KLK6 and KLK7 in OVC cell lines (solid black bars) and normal ovary cell lines (N) was analyzed by qRT-PCR and normalized against a “primary-like” normal ovarian cell line (IOSE523; solid gray bar). IOSE523 cells begin to senesce after twenty passages while FIOSE118 cells are immortal. The mean fold change represents triplicate measurements, and standard error bars are shown.

To pre-screen human genes that have a high potential for use as early detection biomarkers, the BioXM bioinformatics platform was used with query strings including ovarian, biomarker, up-regulation, down-regulation and over-expression. The output data set contained a qualified list of 117 genes (Table 1) that represent diverse processes, including apoptosis, proliferation, invasion, metabolism, and angiogenesis. For mRNA expression screening, a library of 19 OVC and two normal ovarian cell lines was used. The phenotype of normal ovarian cell lines was a mixture of epithelial and fibroblastic (data not shown). Initially, all 117 genes were tested in seven OVC cell lines representing different disease grades and subtypes as well as in two normal ovarian cell lines by qRT-PCR (Figure 1A). In the majority of these cell lines, mRNAs of the clinically established OVC biomarkers CA-125, HE4 and CEA were overexpressed. From the first stage of screening, 30 candidate genes were selected that are differentially expressed in cancer versus control cell lines: *APOD*, *BCHE*, *BCL-2*, *CA-125*, *CDX2, CLDN3, CLDN4, CSF1, DAB2, DUSP1, ETS1, IGFBP5, IL13RA2, JUP, KLK5, KLK6, KLK7, KLK8, KLK13, MAGEA4, MASPIN, MIF, MLANA, MSLN, P11, PRSS8, ST14, TNFRSF1B, VEGFC*, and *WFDC2*. Of these, 12 genes were consistently either up- or down-regulated by more than 10-fold in over 70% of all OVC cell lines that represent different grades, but were mostly derived from late-stage OVC with *BCL-2, CDX2, KLK7, KLK6, P11* and *PRSS8* genes up-regulated, and *IGFBP5, DUSP1, DAB2, VEGFC, IL13RA2* and *APOD* genes down-regulated. Among these 12 genes, *KLK6* and *KLK7* were consistently up-regulated in the majority of cell lines in the OVC cell line library, which was originally created for mRNA expression screening (Figure 1B). A flowchart of the OVC biomarker pre-screening process is provided in Additional file 3.

**Table 1:** **Bioinformatics-based data mining method for the identification of potential ovarian cancer biomarkers**

**Potential biomarkers for ovarian cancer**									
ATP7B	CA125	CLEC3B	**KLK6**	TOP2A	ARID4B	CEA	ID2	IGFBP2	INHA
PDGFA	HE4	**DUSP1**	BSG	CLDN3	REEP5	MIF	IGF2BP1	LGALS3BP	CDC25C
BRCA2	CA72-4	**IL13RA2**	STAT3	CLDN4	CCT3	AFP	IQGAP1	MSLN	NME1
DNAJC15	BARD 1	PLK1	RAET1E	COPS5	CD47	Prolactin	RHOC	ST14	AKT2
KLK14	**BCL2**	VIL2	TITF1	CSF1	ETV4	MUC 1	RNASE2	AMH	ANGPT2
KLK9	IGFII	**APOD**	TFF1	EFNB1	MAGEA4	AMH	SYCP1	CDC25A	XIST
WFDC2	BAG1	CD247	SPINK1	KLK11	SCGB2A1	WT 1	TRIM25	CSF1R	KLK10
ERCC1	BAG3	CDC25B	**PRSS8**	KLK13	SIX5	OGP	**P11**	GADD45A	KLK15
KLK8	BAG 4	**DAB2**	CCNE1	MVP	ZNF217	**CDX2**	CYP2A	HLA-G	KLK5
RBL2	Osteopontn	HMGA1	CEACAM6	PARP1	EYA2	SMRP	PTK2	JUP	**KLK7**
SKP2	Maspin	HOXB7	ETS1	**VEGFC**	ELF1	Bcl-XL	TACC3	MLANA	SOD2
**IGFBP5**	MSN	BCHE	EPHA2	ASNS	MUC5AC	TNFRSF1B			

BioXM software was used to mine the Cancer Gene Index (CGI) to identify genes that are differentially regulated in OVC. This bioinformatics-guided approach identified 117 genes that are supported by experimental evidence in the published literature. Genes are categorized based on signaling pathways and 12 genes showing robust differential expression in the majority of the 21 OVC cell lines versus normal ovarian cell lines (all with p <0.05) are highlighted in bold text.

### Elevated expression of *KLK6* and *KLK7* mRNA was detected in OVC specimens

**Figure 2:**
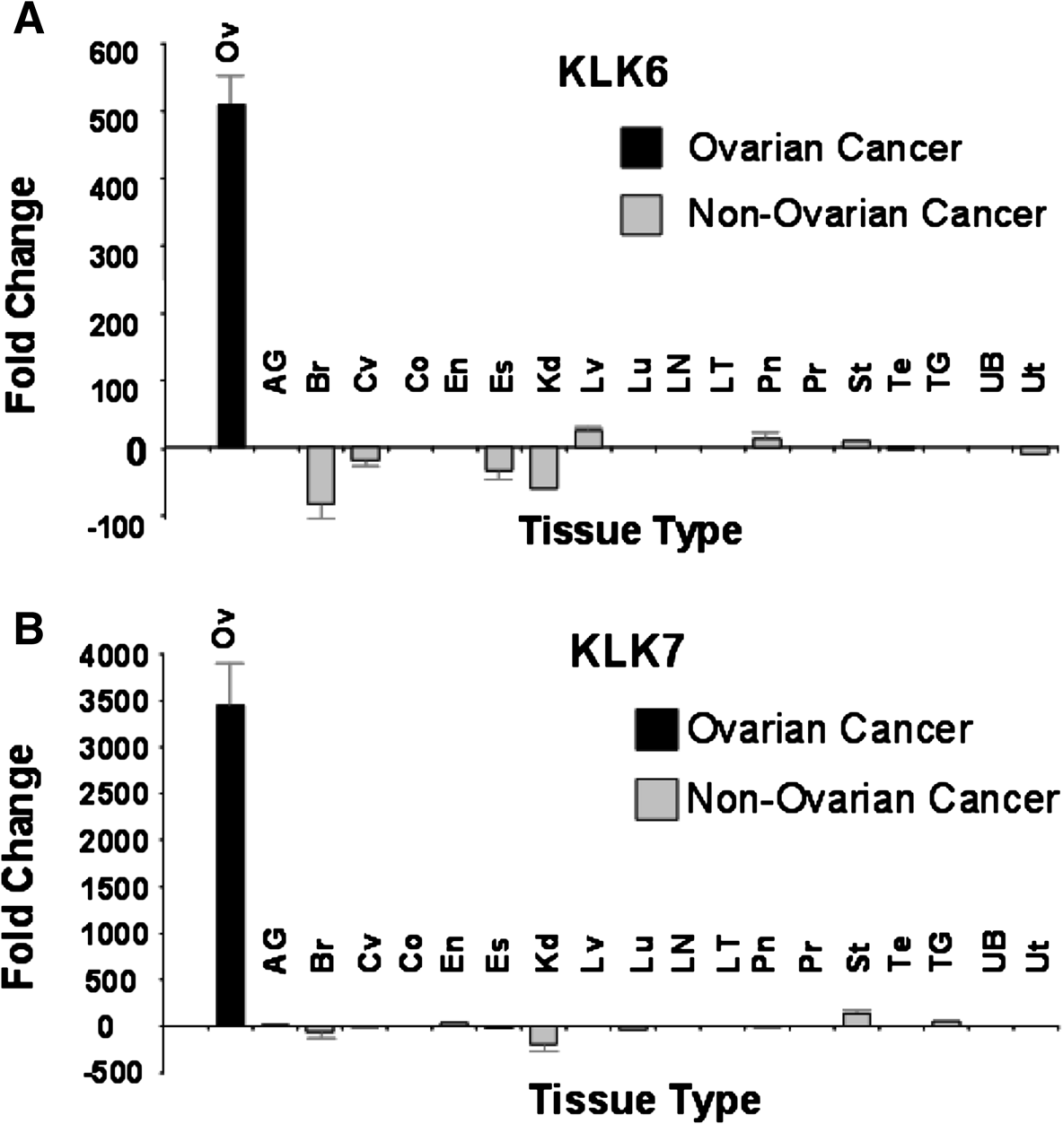
**Differential expression of KLK6 and KLK7 genes is significantly selective for OVC versus 18 other human cancer types.** Over 390 cDNAs derived from 19 different cancer tissues and corresponding normal tissues were assayed by RT-qPCR to quantitatively measure KLK6 **(A)** and KLK7 **(B)** gene expression. The fold change represents the level of gene expression in cancer normalized against the corresponding normal tissue. The mean number of samples used in the assay was 15 for cancer and five for corresponding normal tissues. The statistical significance of differential KLK6 and KLK7 expression in OVC (solid black bar) over other cancer types (solid gray bars) was determined to be p <0.001 (indicated by *) by one-way ANOVA (SigmaStat). Abbreviations for cancer types are Ov = ovarian, AG = Adrenal Gland, Br = Breast, CV = Cervix, Co = Colon, En = Uterine Endometrium, Kd = Kidney, Es = Esophagus, Lv = Liver, Lu = Lung, LN = Lymph Node, LT = Lymphoid Tissue, Pn = Pancreas, Pr = Prostate, St = Stomach, Te = Testis, TG = Thyroid Gland, UB = Urinary Bladder, and Ut = Uterus.

Expression of the 12 selected genes was analyzed by qRT-PCR as a final screening step, with measurements taken in normal and cancer samples from 394 individuals that represented 18 different tumor types apart from OVC. The analysis indicated that the mean differential mRNA expression between ovarian tumor versus normal ovarian tissues was over 500-fold for *KLK6* (p <0.001) and over 3,000-fold for *KLK7* (p <0.001, as indicated by an asterisk). The normal control used here was a mixture of epithelial and stromal components, which represents the true normal ovary. In addition, the differential overexpression of both mRNAs was highly specific to OVC relative to “cancer versus corresponding normal tissues” of other cancer types (p <0.001 at 95% confidence level, CI = 20 with 30% of total population) (Figure 2). Moreover, the difference between cancer versus corresponding normal tissue was the greatest in ovarian cancer compared to other major cancer types. In contrast, the mRNA expression levels of *KLK6* and *KLK7* were down-regulated in breast (87-fold and 75-fold, respectively) and kidney cancers (68-fold and 234-fold, respectively) relative to corresponding normal tissues.

### Expression of *KLK6* and *KLK7* in different subtypes, grades and stages of OVC

**Figure 3:**
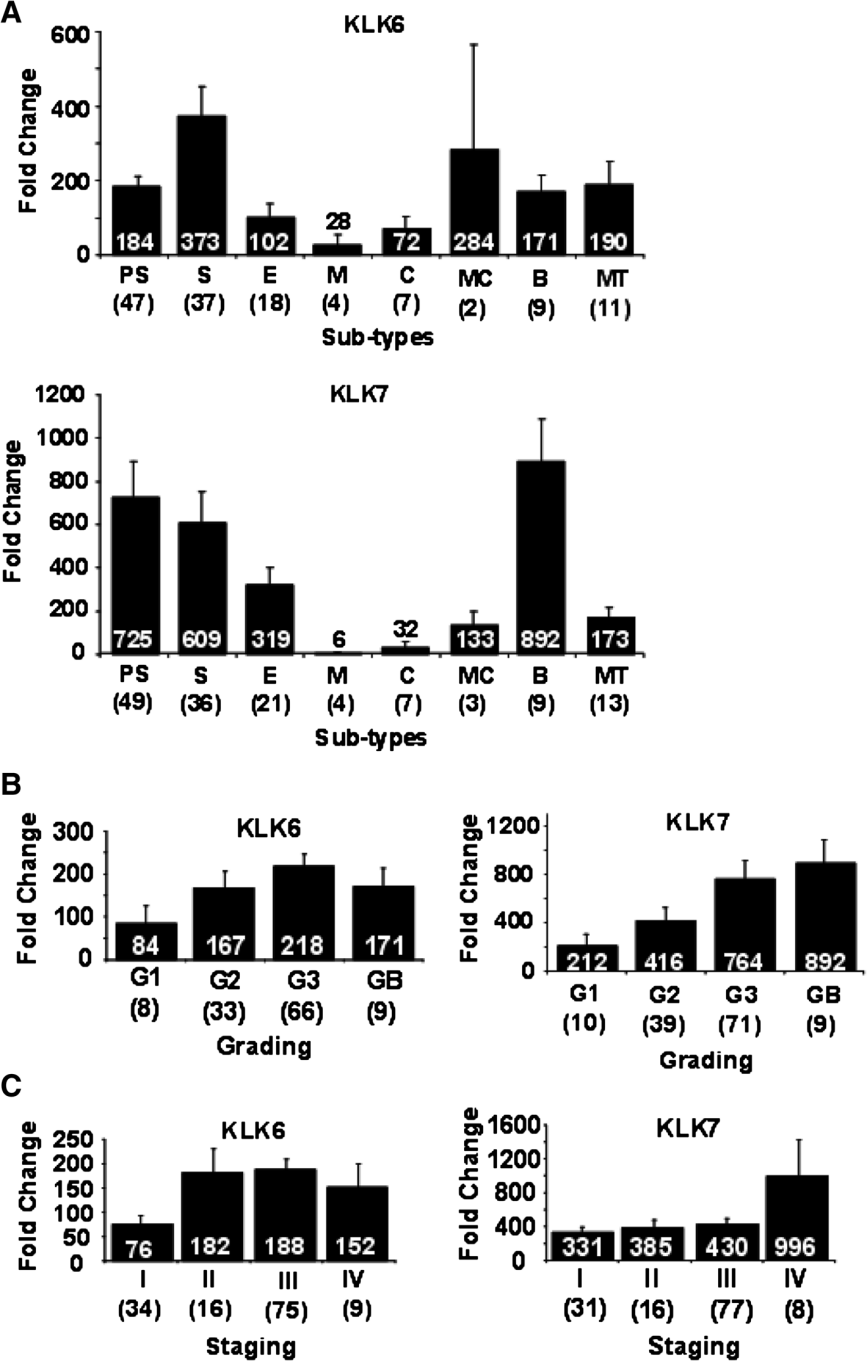
**KLK6 and KLK7 gene expression is subtype-specific (A) and increases progressively in advanced stages (B) and higher grades (C) of ovarian tumors.** Gene expression levels quantitatively measured by qRT-PCR in cDNAs derived from ovarian tumors versus normal ovarian tissue. The fold change level is represented by the height of each histogram and the fold change is indicated in white boldface within the back bars for each subtype (error bars are indicated). Abbreviations for OVC subtypes in **(A)** are PS = Papillary Serous, S = Serous, E = Endometrioid, M = Mucinous, C = Clear Cell, MC = Metastatic Carcinoma (adenocarcinoma of the ovary, metastatic), B = Borderline and MT = Mixed Type. Abbreviations used in **(B)** are G1, G2, and G3, which represent progressive tumor grades; GB is the grade corresponding to borderline subtypes. In **(C)**, the four progressive tumors stages are denoted as I, II, III, and IV. The number of ovarian tumor samples analyzed is given in parentheses.

The gene expression patterns of *KLK6* and *KLK7* in OVCs were analyzed by qRT-PCR in 192 cDNA samples derived from normal (n = 27) and OVC (n = 135 and 142 for *KLK6* and *KLK7*, respectively) tissues representing eight major subtypes of epithelial origin, including papillary serous, serous, endometrioid, mucinous, clear cell, primary metastatic carcinoma and borderline cases. First, subtype-dependent expression of hKLKs was evaluated. Both *KLK6* and *KLK7* were significantly overexpressed (p <0.005) in papillary serous, serous, metastatic carcinoma, borderline and mixed type carcinomas versus normal ovary tissues (Figure 3A). In mucinous and clear cell OVC subtypes, both genes, but especially *KLK6*, were also overexpressed (p <0.01), albeit to a lesser extent compared to the other subtypes. Thus, this overexpression signature was found in subtypes that occur in more than 90% of OVCs, which indicates that *KLK6* and *KLK7* are potential candidates for early detection markers. Next, we evaluated grade-dependent expression and found that *KLK6* and *KLK7* transcripts were overexpressed by 84- and 212-fold, respectively, in low-grade (G1) tumors versus normal controls (p <0.001), and expression of both increased >3-fold from lower (G1) to higher (G3) tumor grades or borderline (GB) tumors (Figure 3B). For tumor staging, both *KLK6* and *KLK7* mRNA levels were elevated 76-fold and 331-fold, respectively, in stage I versus normal controls (p <0.001) (Figure 3C). The expression of *KLK6* and *KLK7* was not statistically significant (p >0.05) between higher grades and stages. These elevated mRNA levels were maintained in advanced tumor grades and stages. Together, these data suggest that *KLK6* and *KLK7* may be very useful as biomarkers for detection of low-grade and early stage OVCs.

### Overexpression and specificity of *KLK6* and *KLK7* in tumor epithelia

**Figure 4:**
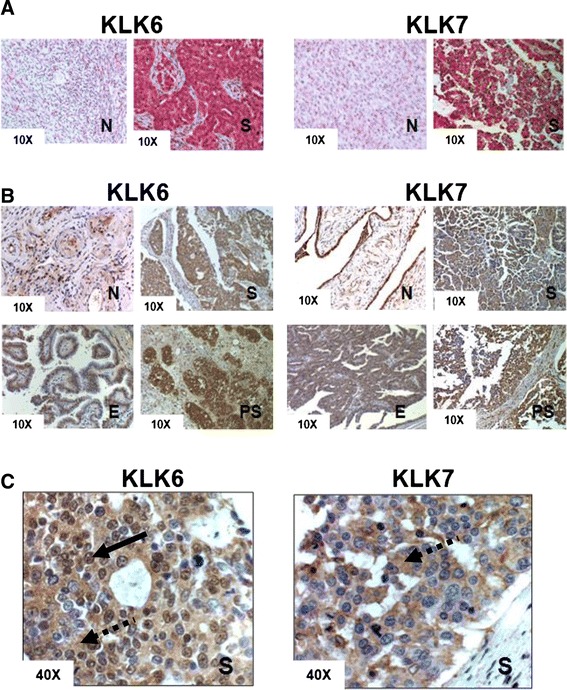
**KLK6 and KLK7 genes and proteins are overexpressed in epithelia but absent in stroma of ovarian tumors. (A)** To detect mRNA in tissues, serous ovarian tumors (S) and normal tissues (N) were hybridized *in situ* with KLK6 and KLK7 oligonucleotide probes followed by visualization of red chromogen staining by bright field microscopy under 10 × magnification. **(B)** To detect KLK6 and KLK7 protein expression in tissues, subtypes of ovarian tumors (S = Serous, PS = Papillary Serous, E = Endometrioid) and normal ovary tissues (N) either in whole mount sections or tissue arrays were analyzed with IHC. DAB staining was visualized by brightfield microscopy. **(C)** Detection of KLK6 and KLK7 protein levels in serous carcinomas by immunohistochemistry, observed under 40 × magnification that allows visualization of nuclear staining, which is indicated by a solid arrow. Cytoplasmic staining is indicated by a dotted arrow.

The overexpression and localization of *KLK6* and *KLK7* were verified by histologic analysis of 512 samples from normal ovary and ovarian tumors, using hybridization of customized oligonucleotide probes for each gene *in situ* in either whole mount or tissue arrays. While *KLK6* and *KLK7* transcripts were expressed at a basal level in normal ovaries, the expression of these two genes increased significantly in tumors versus controls, and their expression was limited exclusively to the epithelium compartment of all ovarian tumors analyzed (Figure 4A). Meanwhile, neighboring tumor stroma, regardless of subtype, was negative for expression in all cases analyzed. Moderate differences in staining intensities were observed between low versus high grade, and early versus late stages of ovarian tumors (data not shown). Immunohistochemical analysis of KLK6 and KLK7 on the same set of tissues demonstrated that protein expression patterns of the KLKs were identical to those seen with *in situ* hybridization (Figure 4B). Both proteins are expressed exclusively in tumor epithelium of serous, endometrioid and papillary serous cancers, whereas the neighboring stroma was minimally positive in all subtypes of ovarian tumors tested (Figure 4B). The KLK7 protein had a predominantly cytoplasmic localization in serous subtypes of OVC cells, while KLK6 showed both cytoplasmic and nuclear staining (Figure 4C).

### The levels of KLK6 and KLK7 mRNA and proteins are elevated in tissue and sera from early stage papillary serous and serous OVC patients

**Figure 5:**
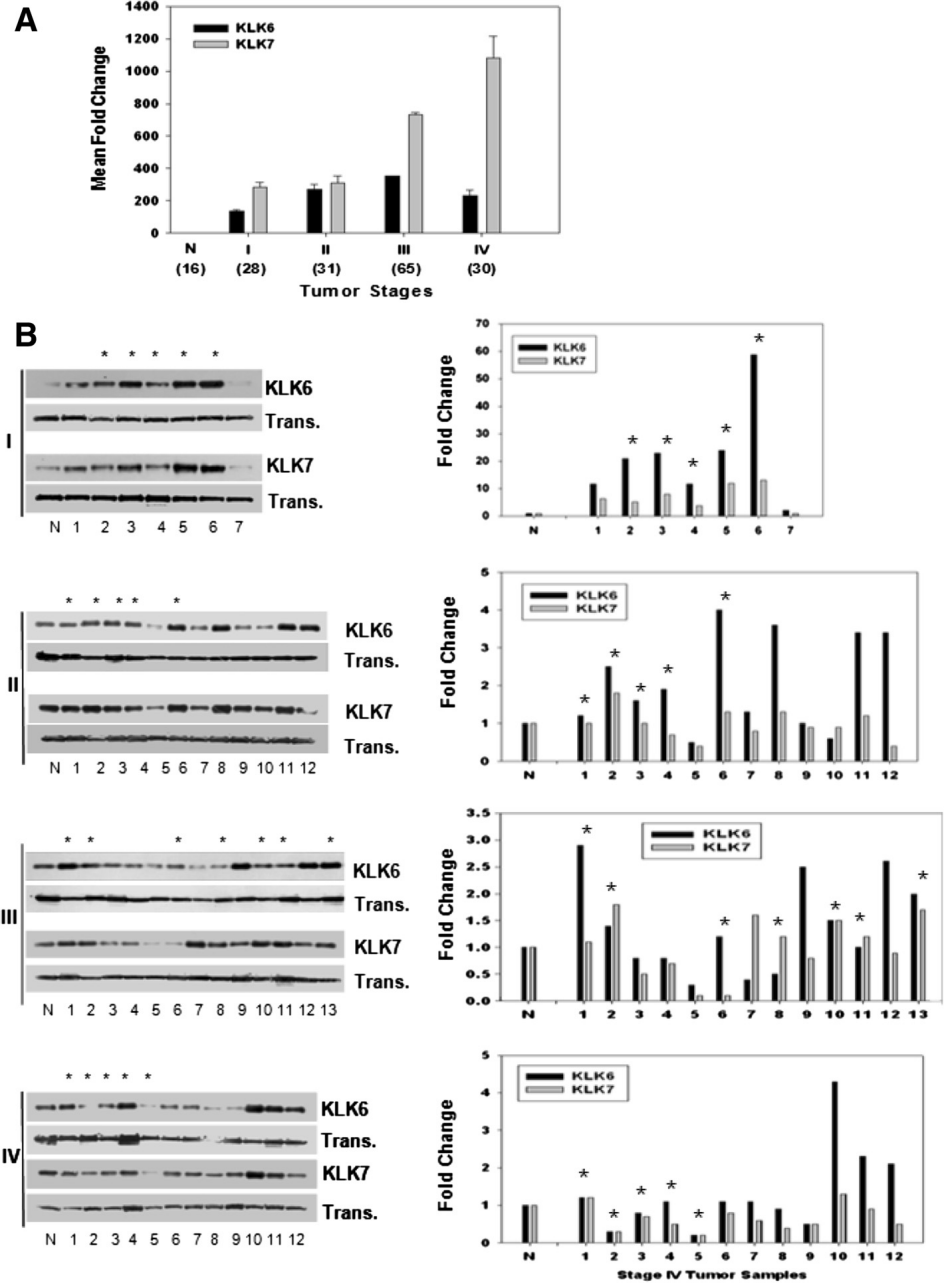
***KLK6*** **and** ***KLK7*** **mRNA and protein levels significantly increase in tissue and serum samples from early stage serous carcinomas. (A)** Gene expression of KLK6 (solid black bar) and KLK7 (solid gray bar) in serous and papillary serous carcinomas from each tumor stage (I, II, III, and IV) was analyzed by qRT-PCR, and the mean fold changes were calculated by normalizing against expression in normal epithelial ovary tissues (N). The number of samples used in the analyses is denoted in parentheses, and standard error bars are indicated. **(B)** Specific protein expression analyzed in immune-blots of pooled normal (N) donor sera (n = 11) and various subtypes (serous and papillary serous only is indicated by an asterisk) from OVC patients of multiple stages (I, II, III, and IV). All serum samples were pre-cleared to deplete the 12 most abundant serum proteins prior to electrophoresis (SDS-PAGE). KLK protein expression in each sample was normalized relative to the internal transferrin signal. The histograms in the graphs adjacent to the immune-blots display the fold changes in KLK protein expression levels, normalized against pooled normal (N) serum and transferrin, as measured by quantitative densitometry.

Elevated KLK expression during the early stages of serous and papillary serous ovarian carcinomas, which comprise the most frequently diagnosed ovarian tumors, was found in an analysis of 59 early stage OVC samples obtained from the TJU and MDACC tissue archives. Our data consistently support that *KLK6* and *KLK7* mRNA expression is elevated in all tumor stages of serous and papillary serous carcinomas relative to normal ovarian epithelial tissues (Figure 5A). Transcripts were over-expressed by nearly 200- to more than 300-fold in stage I or II carcinomas versus normal ovarian tissues (p <0.001 for both KLK6 and KLK7), indicating their utility as excellent biomarkers for early detection of serous and papillary serous subtypes in biopsy samples. In these ovarian tumor subtypes, KLK7 expression, but not that of KLK6, continued to increase in stage III and state IV. Since KLK family members are secreted proteins [52], we further investigated the protein levels by immune-blot analysis of sera obtained from OVC patients. Serum samples were pre-cleared to deplete the most abundant serum proteins (see Materials and methods), and KLK6 and KLK7 did not bind to serum proteins that bound to the pre-clearing column (data not shown). In these pre-cleared samples, KLK6 and KLK7 protein expression was significantly elevated in stage I serous and papillary serous OVC when compared with pooled normal serum (Figure 5B). Other subtypes (no asterisk, and subtypes not indicated) showed mixed expression levels in the serum samples from early stage tumors. Densitometry analysis of serous OVC and these two subtypes in stage I versus pooled normal serum showed a mean fold-increase of 22-fold for KLK6 and 6.7-fold for KLK7 (p <0.01, Figure 5B), suggesting that both proteins may be useful as early detection biomarkers in serum samples.

### KLK6 and KLK7 can complement established OVC markers HE4 and CA-125 for early detection of ovarian cancer

**Figure 6:**
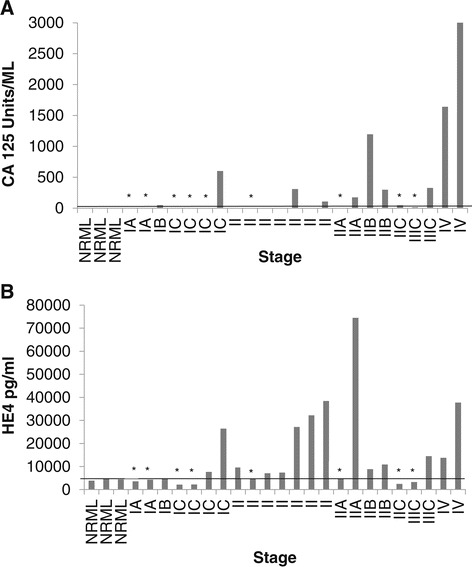
**CA-125 and HE4 levels were both increased in only 58% of serum samples of early stage serous carcinoma patients as measured by ELISA.** CA-125 **(A)** and HE4 **(B)** production was measured by ELISA. Levels of these proteins were calculated as averages of triplicates. The line across the figures is the low threshold for protein up-regulation. An asterisk (*) indicates that this particular patient demonstrated no up-regulation in both CA-125 and HE4.

The most common criteria that clinicians have been using to detect OVC are CA-125 levels, pelvic examination, presence of ascites and family history. Due to the limitations of CA-125 for early detection of OVC, the addition of HE4 as an early detection biomarker improved overall sensitivity, but this combination may still be insufficient for detecting different OVC subtypes. ELISA assays to detect those proteins in our early stage OVC patients (Figure 6) demonstrated that CA-125 was detected at above normal levels in only 7 out of 19 early stage patients and in 3 out of 4 advanced stage (stages IIIC and IV) patients, with the highest overall levels seen in stage IV patients. HE4 was found in 11 out of 19 early stage patients and in 3 out of 4 advanced stage patients where the highest levels were detected in one stage IIA patient, in three stage II patients and in one stage IV patient. These results indicate that for early stage patients (Stages I and II) the sensitivity (or true positive rate) for CA-125 was only 0.61, and 0.7 for HE4. In addition, we defined a group of seven “false-negative” patients for both CA-125 and HE4. This category included 4, 2 and 1 patient with stage I, stage II and stage III disease, respectively. When the same patient population was analyzed for KLK6 and KLK7 expression by immune-blotting, both proteins were found to be significantly up-regulated, particularly in Stage I serous and papillary serous ovarian carcinoma (Figure 5B). In addition, our preliminary KLK7 serum ELISA data indicate that levels of this protein in OVC patients are significantly higher compared to its levels in normal individuals (p <0.05), and there is no significant difference in serum KLK7 levels between benign and normal individuals (P >0.05). An additional figure file demonstrates this in more details (see Additional file 4). Comparison of KLK7 levels between the OVC (n = 17) and the benign (n = 19) groups provided a sensitivity of 62.5% (95% confidence interval [CI] 35.4%-84.8%) and specificity of 94.4% (95% confidence interval [CI] 72.7%-99.9%). When KLK7 levels were compared between the OVC and the normal group (n = 19), sensitivity was calculated as 62.5% (95% confidence interval [CI] 35.4%-84.8%) and specificity calculated as 100% (95% confidence interval [CI] 81.4%-100%). When using area under the receiver operator characteristic curves (AUC ROC) to evaluate KLK7 as a potential biomarker, AUC value was 0.83 (95% confidence interval [CI] 0.69-0.97) when comparing the OVC group to the normal group and 0.73 (95% confidence interval [CI] 0.55-0.91), when comparing the OVC group to the benign group. AUC values can be in the range 0.5 (for a non-informative biomarker) to 1 (for a perfect biomarker). Similarly to our findings, Shan *et al*. reported that tissue KLK6 concentrations were significantly elevated in the OVC group (N = 259) compared to levels in benign (N = 49) and normal (N = 34) groups (P <0.001). No significant difference in KLK6 concentrations was detected between the benign and the normal groups [53]. These data indicate that KLK6 and KLK7 can be very selective biomarkers for early detection of the most common types of OVC, and may efficiently complement CA-125 and HE4 as early detection tumor biomarkers.

Our approach of querying the CGI to identify potential biomarkers for OVC with BioXM software resulted in several candidates for further tests. This cancer gene database, which is manually curated and was originally derived from clinicopathology-based projects (i.e., tumor staging), is in many cases an excellent source of clinically relevant biomarkers for diagnostic use, especially for early cancer detection. Expression pre-screening in OVC cell lines was a practical solution for obtaining broad expression profiles and conserving invaluable patient samples. Systematic expression screening identified robust *KLK6* and *KLK7* mRNA overexpression in the majority of the OVC cell lines tested, and this may be related to up-regulation in response to high levels of steroid hormones in these cells [54]. Despite robust *KLK* mRNA expression detected by qRT-PCR, these mRNAs were up-regulated only 2.3-fold over the control when analyzed by microarray (data not shown), and this result may perhaps be due to the limitation of the physical kinetics of oligonucleotide probe-to-oligonucleotide substrate binding seen with chip-based detection methods. The validation of KLK6 and KLK7 overexpression, specifically in OVC versus other major human cancer types, again indicates that the pre-screening methods used may be highly effective for identifying candidate biomarkers.

The KLK serine proteases are known to be involved in several cancer types, and cancer-specific expression profiles suggest their diagnostic and prognostic values [25]. The KLK6 protein is normally expressed as a pro-enzyme in multiple tissues in adults, and is activated by cleavage by other proteases whereupon it is secreted into biological fluids [55,56]. Mature KLK6 degrades basic constituents of the extracellular matrix and basement membrane in tissues [57]. In advanced OVCs, overexpression of these proteases by transfection was directly associated with shorter disease-free survival and overall survival [58]. In addition, KLK6 overexpression in ovarian cell lines leads to transformation to a malignant cell phenotype [58], and together with osteopontin in the form of free antigens, has been associated with high grade ovarian tumors [59]. The combination of KLK6 and KLK13 overexpression is associated with tumor recurrence [60]. Similarly, KLK7, also known as stratum corneum chymotryptic enzyme, is also over-expressed in human cancers and is secreted into bodily fluids [61,62]. KLK7 overexpression in ovarian carcinoma cells results in the formation of multicellular aggregates and promotes chemo-resistance [63]. However, in contrast to previous studies, in a recent study, multivariate analyses of KLK7 levels determined by immunohistochemistry and ELISA of tumor tissue extracts demonstrated that these levels were significantly correlated with both overall survival and disease free survival [64].

The much higher expression of KLK6 and KLK7 genes in ovarian carcinomas versus normal ovarian tissues (largely stroma elements and germline-derived) suggests that basal expression is low and may be tightly regulated in the epithelium by the presence of hormones and other factors in normal ovaries. A high proliferation rate of ovarian tumor epithelial elements compared to stroma elements during cancer progression would make KLK6 and KLK7 an excellent set of early detection biomarkers because they are significantly overexpressed to levels above that of normal controls in the majority of ovarian tumor subtypes in early stages. Our immune-histologic analysis of normal ovarian tissues demonstrated that these two proteins were exclusively located on the epithelium surface of normal ovarian tissues but not in the neighboring stroma, and the remarkable increase in mRNA and protein expression was directly associated with an expansion of tumor cells in the tumor epithelial compartment as opposed to a normal ovary, which is largely composed of stroma. This signature may be related to the secretion of cytokines, growth factors and steroids as well as the expression of hormone receptors on the ovarian surface epithelium in epithelial OVCs [65]. Similarly, KLKs are more enzymatically active and expressed at higher levels around the ovulation period when the ovaries are stimulated by gonadotrophin [66], suggesting that steroid hormones may play a major role in KLK regulation. Steroid hormone-related signaling has also been found in other cancer types [67-69], and the up-regulation of KLK6 and KLK7 in OVC may thus involve additional cofactors or unique properties of hormonal surges to explain the specificity of the observed overexpression.

In relation to the clinicopathology of tumor progression, maintaining the overexpression of these two proteins has strong implications in metastasis since they enzymatically target several major extracellular proteins such as fibronectin and laminin, as well as other structural proteins related to myelin basic protein, gelatin and casein [70]. The known activity of KLK7 in the desquamation of cornified layers in normal skin may be very similar to its role in metastasis [71].

Typically, the majority of common epithelial ovarian carcinomas will reach malignancy [72], and our data consistently indicate that *KLK6* and *KLK7* mRNA and protein are progressively overexpressed as tumors advanced to malignancy. As opposed to KLK levels in OVC tissue, KLK levels in serum, when measured by immune-blotting, reach a peak at stage I and then decrease in stages III and IV to low basal levels. While the mechanisms underlying this decrease in serum protein levels are not clearly understood, they may involve either a blockade of secretion pathways or loss of epitopes by enhanced proteolysis of KLK. A relevant example for the latter effect is the decline in KLK3 (also known as prostate-specific antigen (PSA)) levels in serum due to enhanced proteolysis that accompanies disease progression [73].

As described previously, CA-125 and HE4 have been commonly used in the clinic as OVC biomarkers. However, it should be noted that although both were approved by the FDA as OVC biomarkers, they are mainly used for disease monitoring. Nonetheless, in the absence of other biomarkers, they are often used as early detection tools or as a part of combined biomarkers kits such as ROMA and OVA1 [74]. Our measurements of these proteins in early stage patients indicated that in 7 out of 19 early stage patients, both CA-125 and HE4 were below normal levels, and therefore did not complement each other efficiently for early detection purposes. Although our population of early stage patients was relatively small (N =19), our results are consistent with larger studies that indicated low sensitivity, mostly for CA-125 and the inability of combined CA-125 and HE4 tests to detect the disease for a large range of ovarian carcinoma subtypes [15]. In addition, we compared serum expression of CA-125 and HE4 as measured by ELISA assays to the production of KLK6 and KLK7 in the same patients as measured by immune-blot analysis. Immunoblotting may be more sensitive than ELISA, and these differences in sensitivity may account for the different results. However, the finding that 3 out of 4 patients (stage III and IV) demonstrated increased levels of both CA-125 and HE4 may indicate that the ELISA assays do have sufficient sensitivity. In addition, one required characteristic of potential biomarkers is the ease of detection in routine tests. Therefore, we have made progress in our laboratory in developing specific ELISA tests that will be able to efficiently measure levels of these proteins in serum samples. Our preliminary ELISA data in larger populations of early stage OVC patients, benign patients and healthy individuals demonstrated up-regulation, mostly of KLK7, in early stage patients. These data were obtained using commercial ELISA kits or anti-KLK antibodies that have been developed in our lab (data not shown). Since KLK6 is aberrantly glycosylated and is subject to heterogeneity [75,76], we recognize the need to develop a specialized ELISA for this protein that will account for potential post-translation modifications and allow accurate identification of its levels in blood serum.

Lastly, we purpose that KLKs may improve diagnostic specificity of OVC. CA125 tends to increase above normal levels in many benign conditions mostly in premenopausal women. Although it demonstrates better specificity than CA125, HE4 has not been approved to be used as an early detection biomarker on its own. Our analysis of KLK6 and KLK7 presence in normal, benign and OVC patients indicated their significant increase in OVC compared to the benign group. In addition, preliminary AUC/ROC analysis demonstrated relatively high values for KLK7 in the OVC group (0.83 against normal and 0.73 against the benign group). Therefore, KLK7 and possibly KLK6 may help address the concern of over-diagnosis of OVC when used together with other established biomarkers.

## Conclusions

Given the high mortality rate of OVC in later stages [77], and the lack of reliable biomarkers for detection of early stage OVC, this study focused on determining whether KLK6 and KLK7 can be used as potential early detection biomarkers. Serous and papillary serous are major epithelial ovarian carcinoma subtypes, and more than 50% will reach malignancy [72]. The key findings of our study readily address the challenge for robust biomarkers that will enable early detection of OVC in the clinic. The observations that *KLK6* and *KLK7* mRNA and protein levels are highly elevated in OVC tissue and serum in early stage OVCs may provide important tools for diagnosis of these diseases. Therefore, detection of higher levels of KLKs in early stage OVC patients can help in making a definitive diagnosis if diagnostic laparoscopy, a minimally-invasive procedure that is safer and simpler than diagnostic laparotomy, was found to be positive. Since we demonstrated that KLK6 and KLK7 are expressed in tissue as well as in serum of early-stage OVC, detecting these proteins in tissue samples obtained through diagnostic laparoscopy can significantly complement the diagnostic power of this surgical procedure.

One of our main goals was to effectively compare the production of KLK6 and KLK7 to more commonly used biomarkers such as CA-125 and HE4. In order to further develop KLK6 and KLK7 as possibly more sensitive detection tools for routine clinical use, it will eventually be critical to explore a multiplex assay platform that would enable the simultaneous measurement of multiple biomarkers in blood. Such tools for early diagnosis will likely contribute to reducing the mortality rate of OVC.

### Consent

Written informed consent was obtained from the patient for the publication of this report and any accompanying images.

## Additional files

Additional file 1:
**Ovarian cell lines that were used in this study.**
Additional file 2:
**A detailed description of assays and samples that were used in this study.**
Additional file 3:
**A flowchart describing OVC biomarkers pre-screening process.**
Additional file 4:
**KLK6 and KLK7 protein expression was analyzed by IHC and positively stained cells were counted manually in OVC (n=38), benign (n=44) and normal individuals (n=42) (A).** All tissues were in tissue array format (Biomax) and all stained tissue cores were visible under 10X magnification by bright field microscopy. KLK6 positive cell counts for normal and benign were similar but both showed significantly lower positive staining than OVC (P<0.05). Similarly, KLK7 positive cell counts for OVC were significantly higher than normal or benign group (p<0.05). Serum levels of KLK7 were measured in cancer (n=17), benign and normal (n=19 in each group) (B). P<0.05 between normal and OVC groups. Red horizontal line indicates mean values.

## Abbreviations

KLK: Kallikrein
HE4: Human epididymis protein 4
CA-125: Cancer antigen 125
OVC: Ovarian cancer
CEACAM5: Carcino-embryonic antigen-related cell adhesion molecule 5
VCAM1: Vascular cell adhesion molecule 1
FDA: Food and Drug Administration
hKLK: Human kallikrein
NCI: National Cancer Institute
CGI: Cancer Gene Index
NLP: The Biomax BioLT ™ Tool
MDACC: MD Anderson Cancer Center
TJU: Thomas Jefferson University
ANOVA: Analysis of variance
DAB: Diaminobenzidine
PFA: Paraformaldehyde
DIG: Digoxigenin
BSA: Bovine serum albumin
SDS-PAGE: Sodium dodecyl sulfate-containing polyacrylamide gels
PMSF: P-methylsulfonylfluoride
HRP: Horse radish peroxidase
ELISA: Enzyme-linked immuno-sorbent assay
