# Supplementary material for: Kallikrein family proteases KLK6 and KLK7 are potential early detection and diagnostic biomarkers for serous and papillary serous ovarian cancer subtypes
Source: J Ovarian Res. 2014 Dec 5;7:109. doi: 10.1186/s13048-014-0109-z (PMC4271347; doi:10.1186/s13048-014-0109-z)
Supplement: Additional file 1: — Ovarian cell lines that were used in this study. [file 13048_2014_109_MOESM1_ESM.pdf]

OVARIAN CELL LINES						
Name	Source	Morphology	Oncogene	Properties	Tumor Histology	REFERENCES
TOV21G	ATCC	Epithelial	p53+ (WT)	Adherent	adenocarcinoma	[31]
TOV112D	ATCC	Epithelial	her2/neu +, p53	Adherent	adenocarcinoma	[32]
OV-90	ATCC	Epithelial	her2/neu +, p53	Adherent	papillary serous adenocarcinoma	[33]
CAOV3	ATCC	Epithelial	FAM123B,STK11, TP53	Adherent	adenocarcinoma	[34]
SKOV3	ATCC	Epithelial	MLH1, CDKN2A, TP53,PIK3CA	Adherent	adenocarcinoma	[35]
PA-1	ATCC	Epithelial	N-ras + (activated)	Adherent	teratocarcinoma	[36]
SW626	ATCC	Epithelial	N/A	Adherent	adenocarcinoma	[37]
ES-2	ATCC	Fibroblast	P glycoprotein	Adherent	clear cell carcinoma	[38]
SKOV-1	Dr. Howell, UCSD	Epithelial	N/A	Adherent	clear cell carcinoma	[39]
IGROV-1	Dr. Howell, UCSD	Epithelial	N/A	Adherent	adenocarcinoma	[40]
HEY	Dr Howell,UCSD	Epithelial	KRAS+BRAF	Adherent	papillary cystadenocarcinoma.	[41]
OV-2008	Dr Howell,UCSD	Epithelial	N/A	Adherent	serous	[42]
A2780	Dr Howell,UCSD	Epithelial	N/A	Adherent	ovarian carcinoma	[43]
UCI-101	Dr. Carpenter, UCI	Epithelial	p-glycoprotein, EGFR	Adherent	papillary adenocarcinoma	[44]
UCI-107	Dr. Carpenter, UCI	Epithelial	N/A	Adherent	papillary adenocarcinoma	[45]
DOV13	Dr. Bast, MD Anderson	Epithelial	N/A	Adherent	ovarian carcinoma	[46]
CSOC882	Dr. Karlan, UCLA	Epithelial	EGFR, HER2,	Adherent	clear cell ovarian carcinoma (Gr 3)	[47]
2774	Dr. Wolff, MD Anderson	Epithelial	N/A	Adherent	Serous cystadenocarcinoma	[48]
BG-1	Dr. Korch, NIH	Epithelial	N/A	Adherent	ovarian carcinoma	[49]
FHIOSE118	Dr. Cheng , MOFFITT	Epithelial	Normal cells	Adherent	normal cell line	[50]
IOSE523	Dr. Auersperg, UBC	Epithelial	Normal cells	Adherent	normal cell line	[51]

Additional file 1 – Ovarian cell lines that were used in this study
