# Supplementary material for: Kallikrein family proteases KLK6 and KLK7 are potential early detection and diagnostic biomarkers for serous and papillary serous ovarian cancer subtypes
Source: J Ovarian Res. 2014 Dec 5;7:109. doi: 10.1186/s13048-014-0109-z (PMC4271347; doi:10.1186/s13048-014-0109-z)
Supplement: Additional file 2: — A detailed description of assays and samples that were used in this study. [file 13048_2014_109_MOESM2_ESM.pdf]

Purpose and assays	Product	supplier	# of arrays/ samples
Measuring KLKs expression on OVC tissue arrays compared to other types of cancer by qRT-PCR (Fig.2)	The TissueScan Cancer Survey Panel	Origene (Rockville, MD)	Over 390 cDNAs from OVC and 18 other types of cancer
Measuring KLKs expression on different subtypes of OVC and in different grades of the disease by qRT-PCR (Fig. 3)	OVC cDNA panel I-IV	Origene (Rockville, MD)	135 samples were evaluated for KLK6 expression and 142 samples were tested for KLK7 expression in 8 subtypes of OVCs. 116 were evaluated for KLK6 and 129 samples were tested for KLK7 expression in tissues taken from patients in 4 different grades of OVC. In addition, 134 samples were tested for KLK6 and 132 samples were evaluated for KLK7 expression in tissues taken from patients in 4 different stages of OVC. Numbers are further specified in parentheses in Fig. 3.
Measuring KLKs expression on OVC and normal ovarian tissue by ISH and IHC (Fig. 4)	1) Whole-mount paraffin-embedded tissues<br>2) Tissue microarrays	1) Proteogenex (Culver City, CA) and the Tissue Bank of Thomas Jefferson University<br>2) US Biomax (Rockville, MD)	1) Tissues from 10 normal and from 51 OVC patients.<br>2) Tissue arrays that contain 12 cases of ovarian cancer and 12 cancer adjacent normal ovary tissue .
Measuring KLKs expression on OVC and normal serum by Western Blotting (Fig. 5)	1) Ovarian tissue samples.<br>2) Serum samples from normal and OVC patients	1) Tissue banks at MD Anderson Cancer Center and Thomas Jefferson University<br>2) Proteogenex (Culver City, CA) and Bioserve (Beltsville, MD)	1) 16 normal ovarian tissue samples and 154 OVC tissues were tested. Numbers are further specified in Fig. 5a<br>2) Serum from 10 normal females and 44 OVC patients were tested
Measuring CA125 and HE4 production by serum ELISA (Fig.6)	Serum samples from normal and OVC patients	Proteogenex (Culver City, CA)	19 serum samples from early stage OVC patients and 3 samples from normal individuals were evaluated

Additional file 2 – A detailed description of assays and samples that were used in this study
