# Supplementary figures and images for: Kallikrein family proteases KLK6 and KLK7 are potential early detection and diagnostic biomarkers for serous and papillary serous ovarian cancer subtypes
Source: J Ovarian Res. 2014 Dec 5;7:109. doi: 10.1186/s13048-014-0109-z (PMC4271347; doi:10.1186/s13048-014-0109-z)

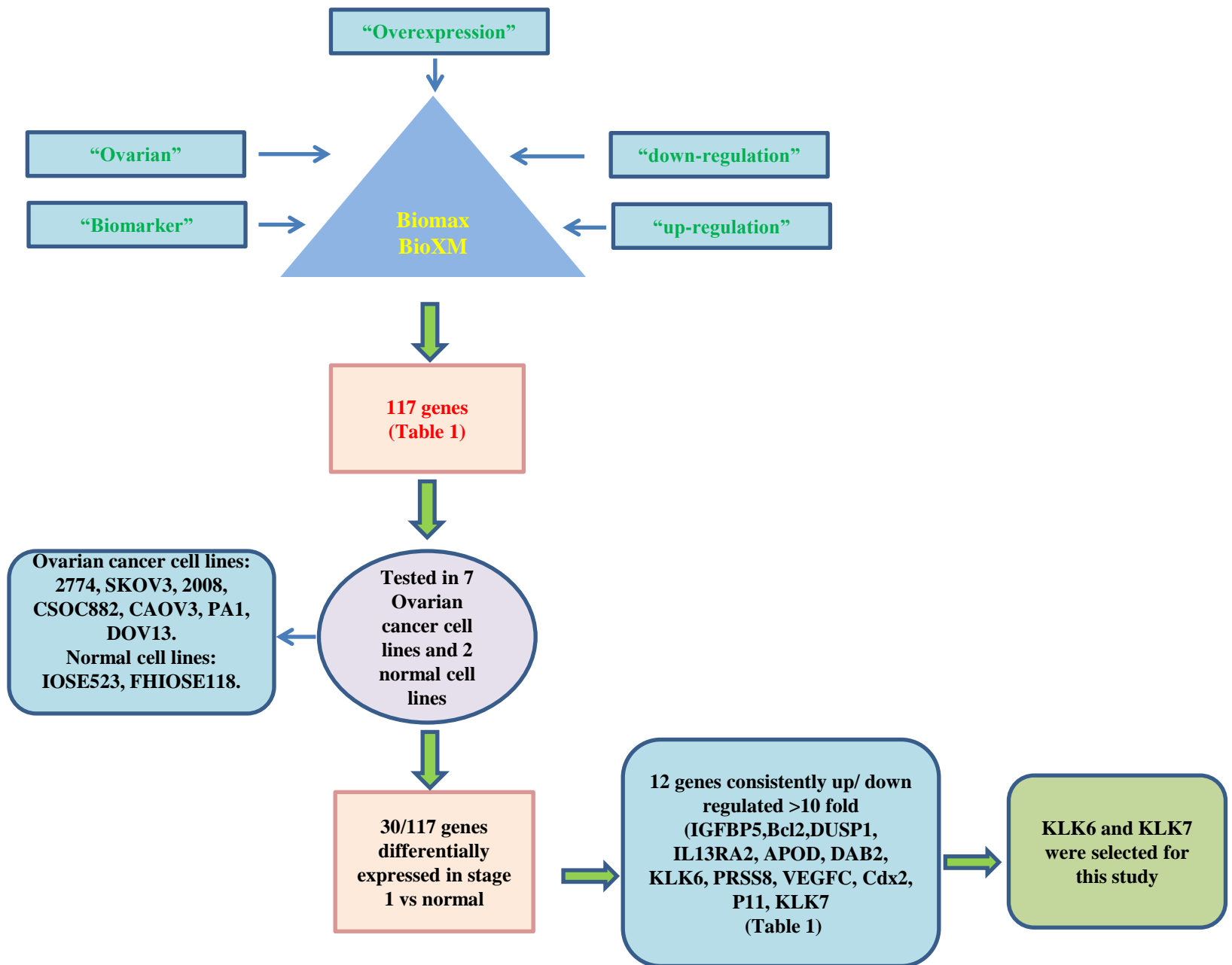

**Additional file 3** – A flowchart describing OVC biomarkers pre-screening process

Supplement: Additional file 3: — A flowchart describing OVC biomarkers pre-screening process. [file 13048_2014_109_MOESM3_ESM.pdf]
