# Supplementary material for: Kallikrein family proteases KLK6 and KLK7 are potential early detection and diagnostic biomarkers for serous and papillary serous ovarian cancer subtypes
Source: J Ovarian Res. 2014 Dec 5;7:109. doi: 10.1186/s13048-014-0109-z (PMC4271347; doi:10.1186/s13048-014-0109-z)
Supplement: Additional file 4: — KLK6 and KLK7 protein expression was analyzed by IHC and positively stained cells were counted manually in OVC (n=38), benign (n=44) and normal individuals (n=42) (A). All tissues were in tissue array format (Biomax) and all stained tissue cores were visible under 10X magnification by bright field microscopy. KLK6 positive cell counts for normal and benign were similar but both showed significantly lower positive staining than OVC (P<0.05). Similarly, KLK7 positive cell counts for OVC were significantly higher than normal or benign group (p<0.05). Serum levels of KLK7 were measured in cancer (n=17), benign and normal (n=19 in each group) (B). P<0.05 between normal and OVC groups. Red horizontal line indicates mean values. [file 13048_2014_109_MOESM4_ESM.pdf]

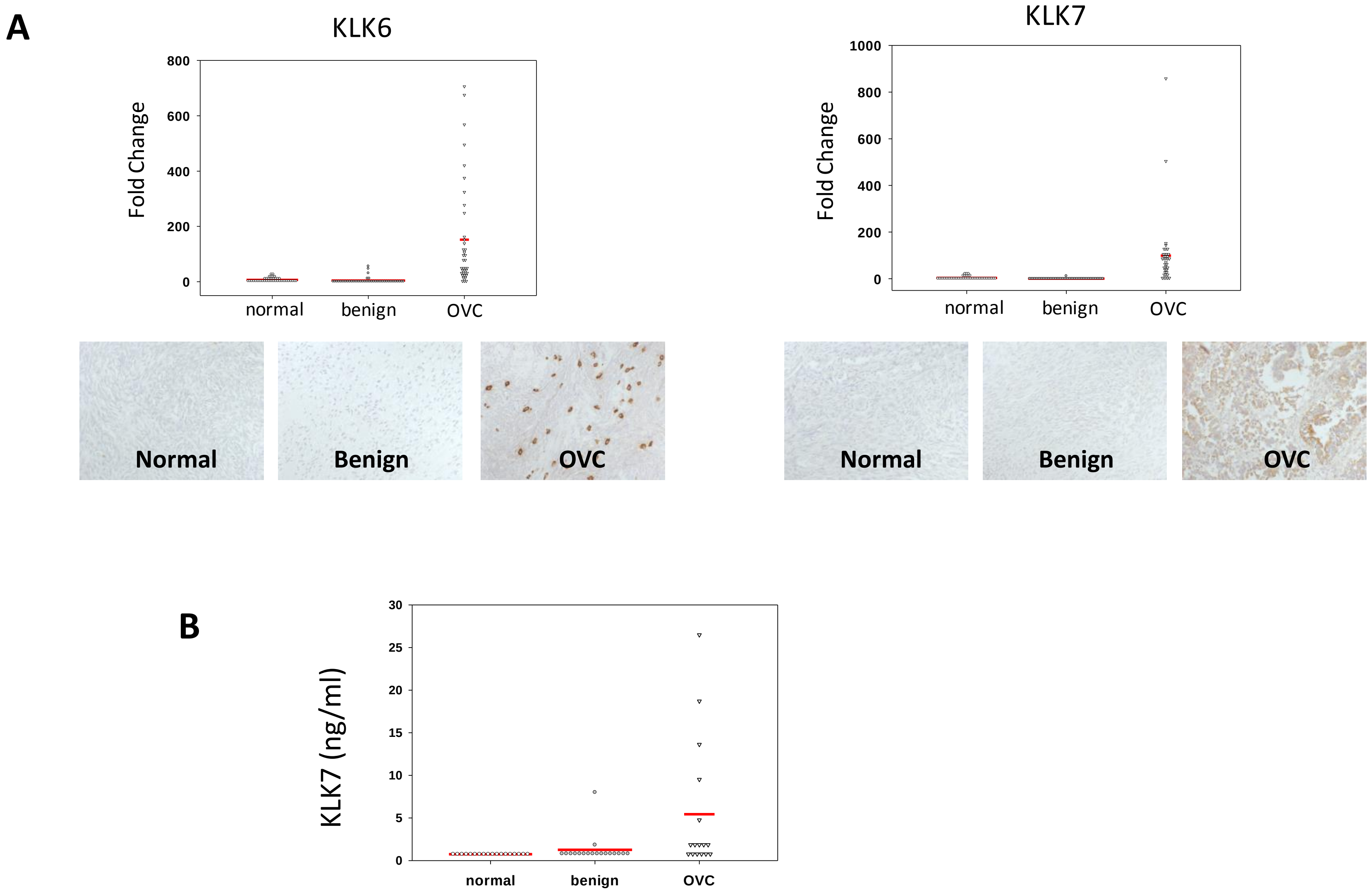

Additional file 4 – KLK6 and KLK7 protein expression was analyzed by IHC and positively stained cells were counted manually in OVC (n=38), benign (n=44) and normal individuals (n=42) (A). All tissues were in tissue array format (Biomax) and all stained tissue cores were visible under 10X magnification by bright field microscopy. KLK6 positive cell counts for normal and benign were similar but both showed significantly lower positive staining than OVC (P<0.05). Similarly, KLK7 positive cell counts for OVC were significantly higher than normal or benign group (p<0.05). Serum levels of KLK7 were measured in cancer (n=17), benign and normal (n=19 in each group) (B). P<0.05 between normal and OVC groups. Red horizontal line indicates mean values.
